# A Novel Tissue Atlas and Online Tool for the Interrogation of Small RNA Expression in Human Tissues and Biofluids

**DOI:** 10.3389/fcell.2022.804164

**Published:** 2022-03-04

**Authors:** Eric Alsop, Bessie Meechoovet, Robert Kitchen, Thadryan Sweeney, Thomas G. Beach, Geidy E. Serrano, Elizabeth Hutchins, Ionita Ghiran, Rebecca Reiman, Michael Syring, Michael Hsieh, Amanda Courtright-Lim, Nedyalka Valkov, Timothy G. Whitsett, Jorge Rakela, Paul Pockros, Joel Rozowsky, Juan Gallego, Matthew J. Huentelman, Ravi Shah, Peter Nakaji, M. Yashar S. Kalani, Louise Laurent, Saumya Das, Kendall Van Keuren-Jensen

**Affiliations:** ^1^ Neurogenomics Division, The Translational Genomics Research Institute, Phoenix, AZ, United States; ^2^ Cardiovascular Research Center, Massachusetts General Hospital and Harvard Medical School, Boston, MA, United States; ^3^ Banner Sun Health Research Institute, Sun City, AZ, United States; ^4^ Department of Medicine, Beth Israel Deaconess Medical Center and Harvard Medical School, Boston, MA, United States; ^5^ Mayo Clinic, Scottsdale, AZ, United States; ^6^ Division of Gastroenterology/Hepatology, Scripps Clinic, La Jolla, CA, United States; ^7^ Department of Molecular Biophysics and Biochemistry, Yale University, New Haven, CT, United States; ^8^ Institute for Behavioral Science, The Feinstein Institute for Medical Research, Manhasset, NY, United States; ^9^ Division of Psychiatry Research, The Zucker Hillside Hospital, Glen Oaks, NY, United States; ^10^ Department of Psychiatry, Zucker School of Medicine at Hofstra/Northwell, Hempstead, NY, United States; ^11^ Department of Neurosurgery, Banner Health, Phoenix, AZ, United States; ^12^ Department of Neurosurgery, St. John Medical Center, Tulsa, OK, United States; ^13^ Department of Obstetrics, Gynecology, and Reproductive Sciences, University of California, San Diego, San Diego, CA, United States

**Keywords:** small RNA, plasma, urine, saliva, cerebrospinal fluid, extracellular vesicle, tissue atlas, extracellular RNA

## Abstract

One promising goal for utilizing the molecular information circulating in biofluids is the discovery of clinically useful biomarkers. Extracellular RNAs (exRNAs) are one of the most diverse classes of molecular cargo, easily assayed by sequencing and with expressions that rapidly change in response to subject status. Despite diverse exRNA cargo, most evaluations from biofluids have focused on small RNA sequencing and analysis, specifically on microRNAs (miRNAs). Another goal of characterizing circulating molecular information, is to correlate expression to injuries associated with specific tissues of origin. Biomarker candidates are often described as being specific, enriched in a particular tissue or associated with a disease process. Likewise, miRNA data is often reported to be specific, enriched for a tissue, without rigorous testing to support the claim. Here we provide a tissue atlas of small RNAs from 30 different tissues and three different blood cell types. We analyzed the tissues for enrichment of small RNA sequences and assessed their expression in biofluids: plasma, cerebrospinal fluid, urine, and saliva. We employed published data sets representing physiological (resting vs. acute exercise) and pathologic states (early- vs. late-stage liver fibrosis, and differential subtypes of stroke) to determine differential tissue-enriched small RNAs. We also developed an online tool that provides information about exRNA sequences found in different biofluids and tissues. The data can be used to better understand the various types of small RNA sequences in different tissues as well as their potential release into biofluids, which should help in the validation or design of biomarker studies.

## Introduction

Extracellular RNAs (exRNAs) have been identified in every biofluid tested to-date (Yáñez-Mó et al., 2015; Yeri et al., 2017; Murillo et al., 2019; Srinivasan et al., 2019; Hulstaert et al., 2020) and are found within extracellular vesicles (EVs) and associated with RNA binding proteins or lipoprotein particles (Valadi et al., 2007; Skog et al., 2008; Arroyo et al., 2011; Turchinovich et al., 2011; Tabet et al., 2014). While longer RNAs (>200 nucleotides) can be detected in biofluids (Wei et al., 2017; Everaert et al., 2019; Hulstaert et al., 2020; Rodosthenous et al., 2020), most studies have focused on small RNA biotypes (<50 nucleotides) (Weiland et al., 2012; Max et al., 2018) for their potential as biomarkers (Witwer, 2015; Nik Mohamed Kamal and Shahidan, 2019). Each biofluid has a distinct small RNA profile. For example, plasma has large amounts of miRNAs, but also high levels of YRNA fragments (Dhahbi et al., 2013; Max et al., 2018; Driedonks et al., 2020). Urine and cerebrospinal fluid (CSF) samples, by comparison, have large numbers of tRNA fragments (tRFs), and smaller numbers of miRNAs (Ben-Dov et al., 2016; Yeri et al., 2017; Godoy et al., 2018; Srinivasan et al., 2019; Tosar and Cayota, 2020). Biofluids also contain measurable piRNA, snoRNA, snRNA, rRNA, fragments of protein-coding genes and lncRNAs found in biofluids (Ishikawa et al., 2017; Yeri et al., 2017; Ferrero et al., 2018; Umu et al., 2018; Akat et al., 2019; Dhahbi et al., 2019; Srinivasan et al., 2019).

These distinct RNA profiles in biofluids suggest coordination of biological processes, such as specific cargo loading into EVs [reviewed in (O’Brien et al., 2020)] and enhanced EV contributions from organs that produce or are more proximally perfused by the biofluid interrogated. While blood cells certainly contribute to RNA patterns found in plasma/serum (Savelyeva et al., 2017; Max et al., 2018), the contributions of specific tissues to acellular biofluids such as urine and CSF are poorly investigated (Cui and Cui, 2020). These are important considerations as many biomarker studies place greater importance on “tissue-specific” miRNAs even though the data used to support these designations are generally poorly defined. Differences in less-studied circulating exRNA biotypes (such as tRNAs/tRFs) have exhibited increased tissue and disease specificity (Magee et al., 2019; Qu et al., 2019; Maleki Dana et al., 2020). For example, the abundance of tRNAs varies temporally and between tissues, and are demonstrated to play a role in a variety of diseases (Dittmar et al., 2006; Sagi et al., 2016; Pan, 2018). piRNAs have also been examined for somatic expression and tissue-enrichment (Martinez et al., 2015; Perera et al., 2019; Chen et al., 2020). While substantial, long RNA tissue atlases have been developed (GTEx, FANTOM, Human Body Atlas), there are fewer, less complete tissue atlases for small RNAs available (Landgraf et al., 2007; Ludwig et al., 2016; Rahman et al., 2020). These tissue atlases for small RNAs have one or more limitations: lack of consistency in analytical technique, insufficient number of replicates per sample type, or samples from disease cases without sufficient numbers of normal controls. Here we describe a tissue atlas of small RNA-seq from postmortem samples from 90 human tissue samples (30 tissues with three biological replicates each) and four biological replicates each of red blood cells (RBCs), monocytes, and peripheral blood mononuclear cells (PBMCs). To make this data uniform, easily accessible and usable to other investigators, we have also processed all of the data with a consistent bioinformatic pipeline, exceRpt (Rozowsky et al., 2019), which assesses all RNA species and fragments that align to the human genome.

Many bioinformatic tools and small RNA databases, such as miRbase, rely on alignment with the canonical sequence for each miRNA or small RNA, which is typically the first discovered sequence and/or the sequence thought to be most abundantly expressed. While consensus sequences are relied upon for naming and organizing RNA biotypes, it is well known that miRNA sequence variation exists, giving rise to isomiRs and diverse RNA fragments (Luciano et al., 2004; Ebhardt et al., 2009; Lee et al., 2010; Martí et al., 2010). IsomiRs are sequences that exhibit slight nucleotide differences (isoforms) from the canonical sequence in miRBase (Guo and Lu, 2010). miRNAs are the most diverse class of small RNA with 1000s of isomiRs that are often not included in downstream analysis, because many analyses pipelines collapse/sum isomiR counts and attribute them to the corresponding canonical mature sequence. Not only are these other isomiRs and RNA fragment sequences measurable, but some are found at higher levels in certain tissues and biofluids than the canonical sequence (Guo and Chen, 2014; Haseeb et al., 2017; Wu et al., 2018). Tissue-dependent expression of isomiRs were found to be biologically active and to target mRNAs in tissues (Londin et al., 2015). Thus, a more comprehensive view of unique RNA sequences is likely to expand the repertoire of disease- and tissue-representative biomarkers (Guo and Chen, 2014; Haseeb et al., 2017; Wu et al., 2018), which in turn may have utility as biofluid-based biomarkers to enable liquid tissue biopsy. For example, in a 2017 paper, Telonis *et al.* described the ability to discriminate between 32 different cancers in TCGA, based solely on the presence and absence of isomiR sequences (Telonis et al., 2017). They found that some isomiR sequences were specific to some tissues and cancers. Telonis went on to apply this approach in breast cancer where they used isomiRs to identify breast cancer subtypes (Telonis et al., 2015). The shifting of a single base can change the targeting of isomiRs for their downstream mRNA target (Karali et al., 2016; Yu et al., 2017).

RNA-sequencing (RNA-Seq) has had a substantial impact on identifying and measuring EV exRNA cargo and has been gaining momentum as a technique for diagnosing and monitoring disease (Van Keuren-Jensen et al., 2014; Byron et al., 2016; Marco-Puche et al., 2019). Many assays require targeting of specific RNA sequences, and thus limit the scope of detected RNAs. Thus, RNA-seq offers not only the ability to monitor a larger number of canonical small RNAs, but also enables monitoring of non-canonical sequences, such as isomiRs, which may be superior discriminators for disease and tissue-enrichment, identifying functional changes, differentiating between disease and health or distinguishing between source tissues. Changes at the 5′ end of isomiRs can change the position of the seed sequence [nucleotides 2-7; (Bartel, 2009)] thereby altering the binding to the target mRNA (Bartel, 2009; Ellwanger et al., 2011), while nucleotides at the 3’ end of the isomiR can also change the stability and targeting of the RNA (Grimson et al., 2007; Agarwal et al., 2015). Although the functions associated with most isomiRs are not known, given the lack of studies in this area, the inclusion of these reproducible sequences increases the number of tissue-enriched targets. It is currently unclear how accurately these slight differences in nucleotides can be measured by qRT-PCR or other methods (Witwer, 2015; Avendaño-Vázquez and Flores-Jasso, 2020).

Here we focused on the use of RNA-seq data to identify individual RNA sequences found in tissues and biofluids. We are aware that there are several advanced adaptations to small RNA sequencing that offer enhanced detection of tRNA fragments with base modifications, mRNA fragments, or sequences with 5′ or 3′ end modifications more comprehensively (Abdelhamid et al., 2014; Cozen et al., 2015; Gogakos et al., 2017; Giraldez et al., 2019; Xu et al., 2019). However, for these experiments, we chose commonly used sequencing kits for their reproducibility, uniformity (Giraldez et al., 2018; Yeri et al., 2018) and their ability to be directly compared across biofluid samples that are already sequenced and readily obtained from several diverse cohorts. We believe that our findings, and the creation of a searchable, online tool, will aid researchers validating their own small RNA studies across tissue types and biofluids.

## Materials and Methods

### Tissue Samples

#### RNA Isolation

Fresh frozen tissue was sliced into pieces that were no greater than 0.5 mm in thickness on dry ice to maintain integrity and placed in 1 ml of RNA Later ICE. The RNA later-Ice immersed tissue incubated for at least 16 h at −20°C to allow for absorption. The tissue was then immediately placed in a glass 16 mm × 100 mm Covaris tube and mixed with 400 μL of cold lysis/binding buffer from the mirVana miRNA Isolation Kit. The Covaris was set to treat each sample at a peak power of 452, a duty factor of 17.4, and cycles/burst of 280. Each sample was then treated for 15–30 s in a Covaris S220 sonicator. Lysate was transferred to a new pre- chilled 2 ml Eppendorf tube with 40 μL of miRNA homogenate solution, and incubated on ice for 3–5 min. Total RNA was extracted according to manufacturer’s protocol with the Total Exosome RNA and Protein isolation kit (cat# 4478545, Thermofisher Scientific, Waltham, MA). RNA was eluted using 100 μL of 95°C ultrapure water. Total RNA was treated with DNase, Turbo DNA-free kit (AM 1907, Thermofisher Scientific, Waltham, MA). DNA-free Total RNA was cleaned and concentrated using Zymo clean and concentrator kit (R1016) and eluted in 28 μL of ultrapure water. RIN values for tissues can be found in Supplementary Table S1.

#### Perkin Elmer Small RNA Library Preparation

Small RNA libraries were generated using NEXTflex Small RNA Library Prep Kit v3 following the manufacturer’s instructions with 250 ng of RNA using 100% adapter and 16 cycles of PCR amplification. Following PCR amplification, libraries between 140 and 160 bp in size were gel purified using 6% TBE gels followed by ethanol precipitation and resuspension in 11 μL of ultra-pure water.

#### Small RNA Library QC and Pooling

Gel purified libraries were quantified using Agilent 2100 Bioanalyzer with High-Sense DNA chips. Equimolar amounts of libraries were pooled and quantified by Bioanalyzer with High-Sense DNA chips. Pooled libraries were normalized and denatured at a working concentration range of 6–8 pM with 5% PhiX spike-in for flow cell cluster generation. Samples were sequenced until new miRNA detection plateaued for each tissue (Supplementary Figure S1).

### Blood Cells

#### Peripheral Blood Mononuclear Cells and Washed Red Blood Cells

Human blood samples were collected with written consent from donors ≥18 years of age under an IRB protocol approved by the Human Research Protections Programs at UCSD. Biofluid samples were labeled with study identifiers.

Whole blood was collected from two male and two female healthy, non-pregnant adult donors, 22–50 years of age. For each donor, blood was collected in the following order: ∼8 ml into a serum BD Vacutainer collection tube (Becton Dickinson, PN 368045) followed by 3 ml × 4.5 ml into CTAD (0.11 M buffered trisodium citrate, 15 M theophylline, 3.7 M adenosine, 0.198 M dipyridamole) collection tubes (Becton Dickinson, PN 367947). The serum tubes were held at room temperature for 20 min prior to centrifugation at 2000 *g* for 5 min with no brake. 500 µL aliquots were transferred from the clear upper serum layer into screw cap 2 ml centrifuge tubes and frozen at −80°C until they were processed. PBMC, platelets, and washed red blood cells (RBC) were purified from the CTAD tubes. Wide-bore pipette tips were used at all relevant steps to reduce cell shearing and lysis.

For PBMCs, the CTAD tubes were centrifuged at 100 x g for 20 min with no brake and all but ∼100 µL of the supernatant was removed and discarded. For the PBMCs, the remaining supernatant, buffy coat, and a small portion of the RBCs were transferred from the CTAD tubes into a fresh 15 ml tube. Freshly prepared Prostaglandin I2 (PGI2) (Abcam, ab120912-1 mg) was added to ∼2 mM final concentration. The material was gently inverted several times to mix and centrifuged at 100 x g for 20 min with no brake. The supernatant material was removed to near completion and the pellet was mixed in 10 ml of RBC lysis buffer (150 mM NH4Cl, 10 mM NaHCO3, 1.27 mM EDTA) placed at room temperature for 20 min. The material was centrifuged at 500 x g for 5 min and the supernatant was discarded. The pellet was washed twice with 10 ml of Dulbecco’s phosphate-buffered saline (DPBS) each time and centrifuged as before. The pellet was gently resuspended in 2 ml of DPBS and the material was transferred to a 2 ml centrifuge tube and centrifuged as before. The supernatant was carefully removed and the pellet material in the tube was placed at −80°C until processed.

The remaining RBCs within the CTAD tubes were transferred into a 50 ml conical tube and DPBS was added to 50 ml. The cells were centrifuged at 500 x g for 5 min with no brake and the supernatant was decanted. This washing process was repeated two more times. 200 µL aliquots of the remaining washed RBC pellet were transferred into 2 ml screw cap tubes and stored at −80°C until processed.

#### Monocytes

Human peripheral blood was obtained from health adult volunteers in accordance with the guidelines of the IRB of Beth Israel Deaconess Medical Center after informed consent was obtained in accordance with the Declaration of Helsinki. Ten ml of blood from healthy donors were collected via cubital venipuncture into a syringe prefilled with 2.3 ml of 6% Dextran 500 (Sigma-Aldrich, St. Louis, MO) and 1 ml of 3.2% Sodium Citrate (Sigma-Aldrich). After gentle mixing the blood was sedimented for 45 min with the syringe’s nozzle up. The RBC-free fraction was washed once by centrifugation at 2000 *g* for 10 min. The resulting pellet was resuspended in 0.5 ml of HBSS^++^.

The cells were sorted using a Becton Dickinson FACSAria IIu cell sorter equipped with five lasers (350, 405, 488, 561, and 640 nm). The cell populations were sorted through a 70 μm nozzle tip at a sheath pressure of 70 psi and a drop drive frequency of 90–95 kHz. A highly pure sorting modality (4-way purity sorting for FACS Aria, Masks at 0-32-0) was chosen for cell sorting. The flow rate was maintained at an approximate speed of 10,000 events/second. Monocytes were gated based on FSC/SSC properties. The FSC values are proportional to the diameter of the interrogated cells, whereas the SSC values provide information about the internal complexity of the interrogated cell or its granularity. Sorted cells were collected in 5 ml polypropylene tubes containing 1 ml collection medium (RPMI supplemented with 50% FBS, 100 μg/ml gentamicin, 4 mM L-glutamine, 20 mM HEPES) and stored at −80°C until processed.

### Biofluid Samples

#### Collection

The cell-free plasma, urine and saliva samples were from a previously published study (Yeri et al., 2017). Cell-free RNA isolations methods were optimized and compared to identify the best return of small RNAs from biofluid samples (Burgos et al., 2013; Burgos and Van Keuren-Jensen, 2014). Samples were collected from male college athletes ages 18–25. All human subjects provided written consent form prior to enrollment. All samples were collected with consent and approval from the Western Institutional Review Board (WIRB) study ID# 1307009395, dbGaP accession phs001258. Blood samples were collected in EDTA tubes and placed with ice packs until they were processed within 2–3 h of blood draw. Samples were spun for 10 min at 700 x g at 4°C in an Eppendorf 5804 R centrifuge using an A-4-44 rotor to remove cells and debris. The supernatant (plasma) was collected, and stored in 1 ml aliquots at −80°C for RNA isolation and sequencing. Urine was collected in sterile cups and placed in a cooler with ice packs and processed within 2–3 h of collection. Samples were spun at 1900 x g for 10 min at 4°C and 15 ml were pipetted into a 50 ml conical tube for storage at −80°C. Saliva samples were collected by passive drool to collect into a 50 ml conical tube. The sample was spun at 1900 x g for 10 min at 4°C, and 1 ml volumes were placed into 2  ml microcentrifuge tubes and stored at −80°C.

The CSF-EV and plasma-EV samples were collected under the approval of the Institutional Review Board of the North Shore-Long Island Jewish Health System. Lumbar puncture, performed using a standard technique with a 25-gauge, Whitacre point spinal needle after subcutaneous lidocaine was applied. The procedure was conducted with patient sitting up and all procedures took place at 2 p.m. local time. 15–25 ml of CSF was obtained from each subject. The first 2–3 ml obtained after collecting the fluid were sent for routine testing (cell count, proteins, glucose, and VDRL). CSF samples with macroscopic blood as consequence of traumatic procedures were not included in these analyses. A blood draw to obtain 10 ml of peripheral blood into an EDTA tube was also obtained prior to the lumbar puncture procedure. Both CSF and blood samples were centrifuged at 2,000 x g for 10 min at 4°C. Both sample types were flash frozen with liquid nitrogen and stored at −80°C at the biorepository of the Feinstein Institute for Medical Research.

### RNA Isolation

#### CSF-EV and Plasma-EV Samples RNA Extraction

We followed the protocol in the Qiagen exoRNeasy Serum/Plasma kit (Cat No./ID: 77044) to capture extracellular vesicles on an affinity membrane, and then to lyse the vesicles and isolate the extracellular RNA. Briefly, 1 ml plasma samples were thawed at room temperature and centrifuged at 3,000 x g for 5 min at 4°C to remove debris (in an Eppendorf 5804 R centrifuge using an A-4-44). Samples were then applied to the column following the manufacturer instructions. This kit and the EVs isolated have been previously characterized (Enderle et al., 2015; Tang et al., 2017; Srinivasan et al., 2019; Kalani et al., 2020).

#### Cell-Free Plasma, Saliva, Urine RNA Isolation

The samples used were from a previously published study (Yeri et al., 2017). Plasma and saliva samples were isolated (1 ml) using the mirVana miRNA Isolation Kit (ThermoFisher Scientific, Waltham, MA, AM1560) according to (Burgos et al., 2013). Samples were DNase treated using TURBO DNA-free Kit (ThermoFisher Scientific, Waltham, MA, AM 1907). Samples were then cleaned and concentrated using Zymo RNA Clean and Concentrator (Zymo Research, Irvine, CA, R1016) using Protocol: Purification of small and large RNAs into separate fractions and combining the fractions at the end. Urine samples (15 ml) were isolated with Urine Total RNA Purification Maxi Kit, Slurry Format (Norgen Biotek Corp., Thorold, ON, Canada, Cat#29600). Samples were DNase treated on column using RNase-Free DNase Set (Qiagen, Germantown, MD, cat# 79254). Samples were concentrated by Speed Vacuum.

### Sample Preparation for Sequencing

#### CSF-EV and Plasma-EV Samples Small RNA Library Preparation

Small RNA libraries were generated using NEXTflex Small RNA Library Prep Kit v2 (BIOO) with 16 cycles of PCR amplification. Amplified libraries were resolved on a 6% TBE gel for size selection. The 140- to 160-nucleotide bands corresponding to adapter-ligated libraries were excised and recovered in a DNA elution buffer followed by ethanol precipitation and resuspension in 11 μL of ultra-pure water. Gel purified libraries were quantified using Agilent 2100 Bioanalyzer with High-Sense DNA chips. Equimolar amounts of libraries were pooled and quantified by Bioanalyzer with High-Sense DNA chips. Pooled libraries were normalized and denatured at a working concentration range of 6–8 pM with 5% PhiX spike-in for flow cell cluster generation.

#### Cell-Free Plasma, Saliva, Urine Sequencing

The samples employed were previously published (Yeri et al., 2017). Plasma, saliva and urine RNAs were quantified in triplicate using Quant-iT Ribogreen RNA Assay kit, Low-Range protocol (R11490; ThermoFisher). The Illumina small RNA TruSeq kit (RS-200–0048; Illumina) was used for sequencing all samples. RNA input for plasma and saliva was 10–20 ng for all samples and the RNA input for urine was 30 ng for all samples. The reagents from the Illumina TruSeq kit were halved (Burgos et al., 2013). Samples were assigned and unique index. 16 PCR cycles were used for all samples. Samples were pooled and placed on Illumina V3 single read flowcells (GD-401-3001; Illumina).

#### Liver Fibrosis Samples

The samples employed were previously published (Van Keuren‐Jensen et al., 2016). All patients were consented at the Mayo and Scripps Clinics and the Institutional Review Boards of both sites approved the collection of the samples. For liver and serum samples, the miRVana kit (AM1560; Life Technologies) was used according to the manufacturer’s protocol with a slight modification for two phenol chloroform extractions (Burgos et al., 2013). TruSeq small RNA Library preparation Kit (Illumina RS‐200‐0048) was used. Kit reagents were used in a half reaction. Each sample was assigned one of 48 indices. 200 ng of RNA were used from tissue samples, with 15 PCR cycles. The total RNA isolated from each serum sample volume (∼1 ml) was used for small RNA sequencing. Samples were pooled and placed on a single read Illumina V3 flowcell (GD‐401‐3001). One lane of the flowcell was loaded with PhiX as a reference lane to help with low nucleotide diversity in microRNA.

#### Exercise Samples

The samples employed were previously published (Shah et al., 2017). All subjects provided written informed consent under approved Institutional Review Board protocols at the Beth Israel Deaconess Medical Center and Massachusetts General Hospital. Plasma samples (1 ml) were isolated using a modified mirVana PARIS protocol (AM1556, Life Technologies) with sequential phenol-chloroform extractions (Burgos et al., 2013). RNA was cleaned and concentrated (R1016, Zymo Research, Irvine, CA). Sample preparation was done using the Illumina small RNA TruSeq kit (RS-200-0048, Illumina). The kit reagents were halved at all steps with 16 rounds of PCR amplification. Libraires were quantified with the Agilent High Sensitivity DNA Kit (5067-4626, Agilent) and sequenced on an Illumina flowcell.

#### Stroke Samples

The samples employed were previously published (Kalani et al., 2020). This study was approved by the institutional review boards of St. Joseph’s Hospital and Medical Centre. Blood was collected in EDTA tubes. Extracellular vesicles were isolated from the plasma samples using the exoRNeasy Serum/Plasma kit from Qiagen. We followed the protocol in the Qiagen exoRNeasy Serum/Plasma kit (Cat No./ID: 77044) including a centrifugation at 3000 g for 5 min at 4°C to remove debris, after thawing and before RNA isolation. A total of 10 ng of isolated RNA was used in sample preparation (Quant-iT Ribogreen RNA Assay kit, Low-Range protocol (R11490; ThermoFisher). Samples were sequenced as described above for the liver fibrosis and exercise samples, with 16 PCR cycles.

### Data Analysis

#### Processing

All samples were processed using the exceRpt small RNA pipeline (Rozowsky et al., 2019). The gene count tables for miRNAs, tRFs, piRNAs, protein coding genes and yRNAs were taken directly from the summary tables generated by exceRpt. To generate the count tables of sequences in each sample, the adapter trimmed fastq files were first compared against the human genome mapped BAM files from exceRpt to find reads which mapped to the human genome. The genome mapped reads where then identified in the transcriptome mapped BAM file to identify the biotypes of the sequence. Since multi-mappers are common, biotype was assigned in the same priority order as the exceRpt pipeline: miRNA, YRNA, tRNA, piRNA, protein coding, followed by a bin of any other biotype in the annotation. Reads with the same sequence (after adapter trimming) were then collapsed into a counts table with biotype and gene annotations. All counts tables (both genes and sequences) were then loaded into R (v. 4.1.0) for further analysis. Rarefaction curves were generated in R using the vegan package (https://CRAN.R-project.org/package=vegan) to assess sequencing saturation.

#### Tissue Atlas

The count tables from the tissue atlas samples were filtered in R to only genes and sequences present at >25 read counts in >2 of the 3 biological replicates. This allowed us to both include sequences which were well expressed while not removing sequences which were specific to a single tissue type. After filtering, read counts were normalized using median of ratios in DESeq2 (Love et al., 2014). Normalized counts were using to generate diversity and counts boxplots for each tissue type and a UMAP of all sequences was generated using the uwot package in R (https://CRAN.R-project.org/package=uwot). Tissue-enriched genes and sequences were determined using the TissueEnrich package in R with the foldChangeThreshold set to 7 (Jain and Tuteja, 2019). Sequences with specificity of “All” were removed, leaving a table of tissue-enriched, group-enriched and tissue-enhanced sequences and genes. Genes and sequences are assigned using the algorithm from the Human Protein Atlas (Uhlén et al., 2015) and can be grouped as follows: 1) Tissue-Enriched: Genes with an expression level greater than 1 normalized count that also have at least five-fold higher expression levels in a single tissue compared to all other tissues; 2) Group-Enriched: Genes with an expression level greater than 1 normalized count that also have at least five-fold higher expression levels in a group of four tissues compared to all other tissues; 3) Tissue-Enhanced: Genes with an expression level greater than 1 normalized count that also have at least five-fold higher expression levels in a single tissue compared to the average levels in all other tissues, and that are not considered Tissue-Enriched or Group-Enriched. Throughout the paper, these three categories were combined and termed; tissue-elevated.

#### Biofluids

The count tables from the various projects above were processed in a consistent manner. All were loaded into R as counts matrices of genes or sequences. Stacked bar plots of biofluid genome mapping and biotype diversity was generated from the raw count matrices. For further analysis, each biofluid type was filtering independently with a gene or sequence requiring >10 read counts in >50% of the samples for inclusion. As with the tissue atlas samples, filtered count matrices were normalized using median of ratios in DESeq2. Tissue-elevated sequences in biofluids were found based on the overlap of filtered sequences in biofluids samples with tissue-elevated sequences identified above.

#### Diversity Analysis

We use the term gene diversity in tissues to refer to the traditional analysis of collapsing all detected isoforms of a gene to a single parent gene and counting only the parent. We use the term “sequence diversity” as the total number of unique sequences with a normalized expression >10 in any tissue, without collapsing to the canonical parent gene. Similarly, in biofluids, gene diversity was determined by collapsing all isoforms of a gene to the canonical mature sequence and allowing them to count for that gene, sequence diversity in biofluids were determined by summing the number of detected sequences with normalized counts >10 in a biofluid and allowing them to be counted independently. Sequences detected in the biofluid samples with >10 read counts in 50% of the samples from that biofluid, and were listed as tissue-enriched, tissue-enhanced or group-enriched – combined they are designated tissue-elevated. Sequences that were elevated in multiple tissues were counted for each tissue.

#### Differential Expression Analysis

For analyses of liver fibrosis, exercise and stroke, all biofluid samples were loaded into R, filtered as above, and analyzed for differentially expressed sequences using DESeq2. Volcano plots of differentially expressed sequences were generated and colored based on the RNA biotype of the sequences. Differentially expressed sequences (Benjamini-Hochberg p-adjusted values <0.05) were compared to tissue elevated sequences and one sequence was highlighted in a bar graph displaying expression levels.

## Results

### Small RNA Profiles Differ Across Biofluids

**FIGURE 1:**
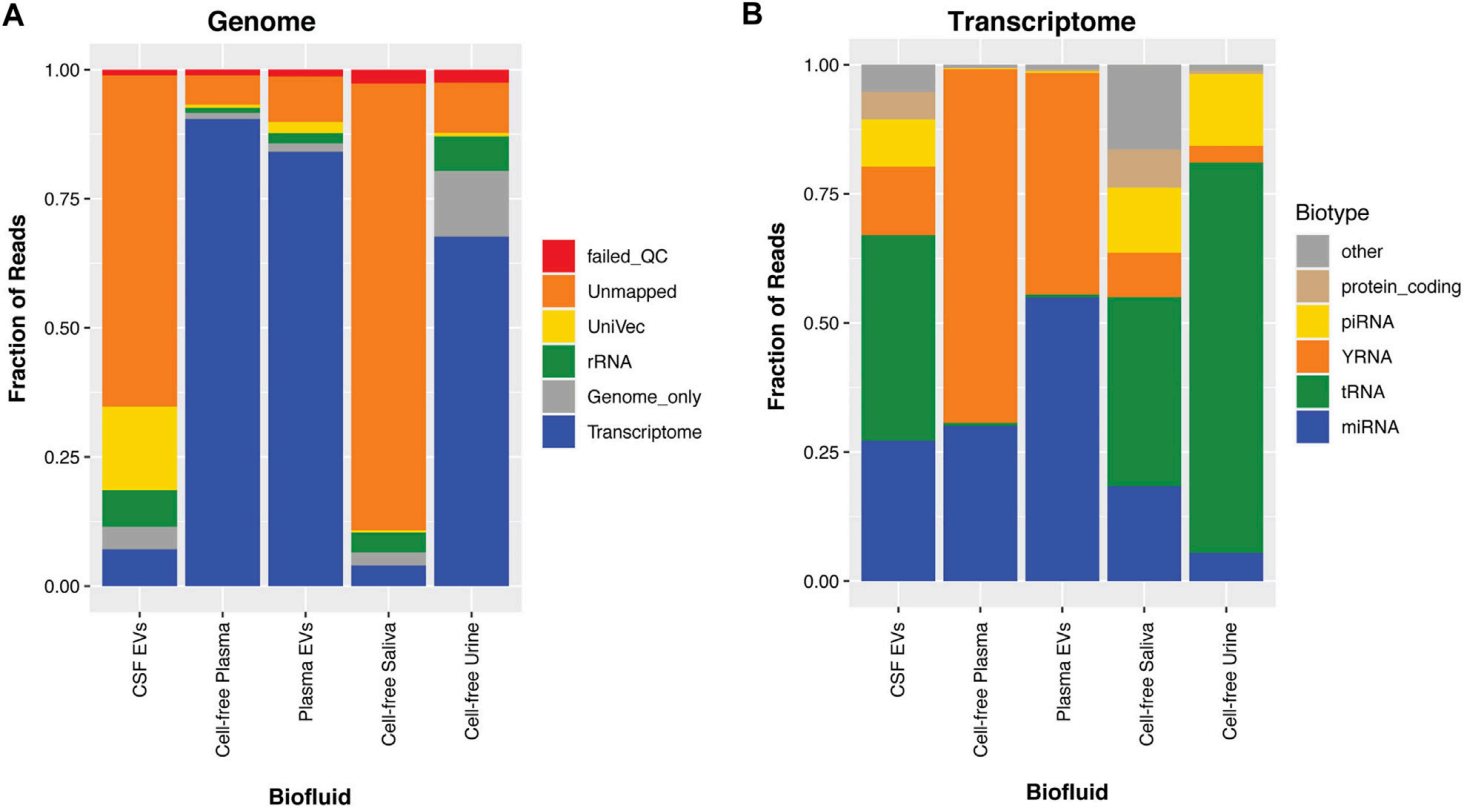
Biofluid Profiles. For the biofluids, CSF-EVs, cell-free plasma, plasma-EVs, cell-free saliva and cell-free urine, the associated small RNAs were sequenced and aligned to the human genome **(A)**. In Panel **(A)**, the reads are aligned to the transcriptome, genome, rRNA, UniVec (laboratory contaminants), are categorized as unmapped, or reads that fail QC. In Panel **(B)**, the transcriptome mapped reads are taken out and further broken down into small RNA biotypes; miRNA, tRNA, yRNA, piRNA, protein-coding fragments, and other (these are categories of RNA that annotate in GENCODE, lncRNA fragments, pseudogenes, etc).

We, and others, have found that each biofluid contains a distinct small RNA profile (ref 20; 1, 3), as can be observed in the percentage of reads mapped to the genome (Figure 1A) or transcriptome (Figure 1B). For this paper, we compare data from a previously published cohort: 179 cell-free plasma samples, 44 cell-free saliva samples, and 203 cell-free urine samples (Yeri et al., 2017). We also included 37 CSF and 35 matched plasma samples isolated with exoRNeasy to enrich for exRNAs contained in EVs.

We show that plasma and urine samples show the highest mapping rates to the human genome, while saliva and CSF samples have lower mapping rates (Figure 1A). CSF typically has very little measurable exRNA (Hulstaert et al., 2020; Burgos et al., 2013; Kopkova et al., 2018; Saugstad et al., 2017; Waller et al., 2017). These low-level RNA inputs make these samples more susceptible to amplification of synthetic (adapter) oligos and common laboratory contaminants (UniVec). Taking the portion of the CSF that maps to the transcriptome (Figure 1B), many of the exRNAs found in CSF are miRNA and tRNA, with >25% going to yRNA, piRNA and protein-coding fragments (Hulstaert et al., 2020; Godoy et al., 2018). Saliva contains high RNA concentrations, but a large fraction of this RNA is not human, with significant contributions from bacterial sequences (Li et al., 2018; Takeshita et al., 2016). Of the reads that map to the human transcriptome (Figure 1B), cell-free plasma is dominated by miRNA and yRNA fragments (yRFs), and EVs enriched from plasma samples have slightly higher levels of mapped miRNA. Finally, urine has low miRNA content, with high levels of tRFs as previously reported (Yeri et al., 2017; Godoy et al., 2018; Srinivasan et al., 2019).

### Generation of a Tissue Atlas of Small RNAs

**FIGURE 2:**
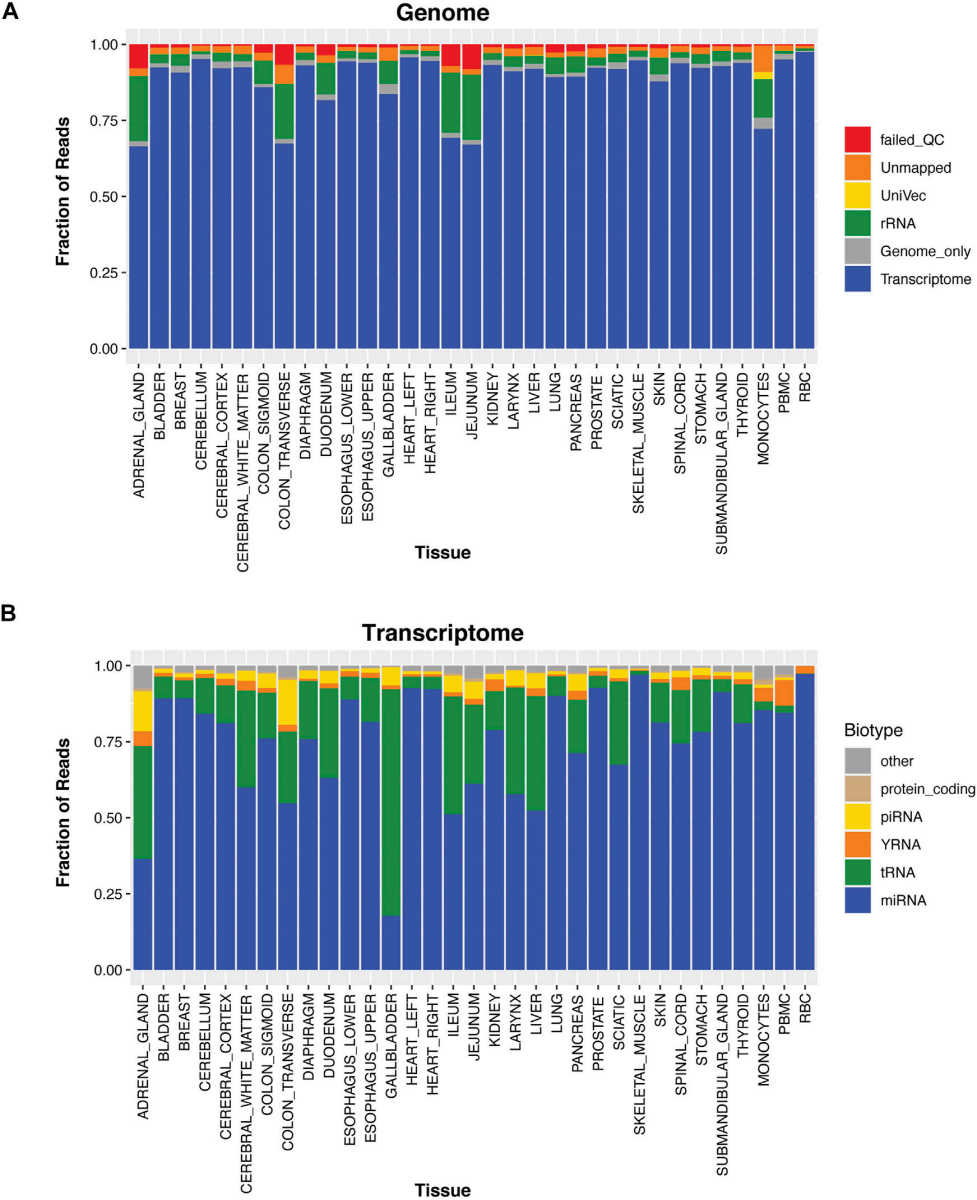
Tissue Profiles. For thirty different tissues, PBMCs, monocytes, and red blood cells we isolated the RNA and sequenced their small RNA contents. In Panel **(A)**, the reads are aligned to the transcriptome, genome, rRNA, UniVec (contaminants), are unmapped, or reads that fail QC. In Panel **(B)**, the transcriptome mapped reads are removed and further broken down into small RNA biotypes; miRNA, tRNA, yRNA, piRNA, protein-coding fragments, and other (these are categories of RNA that annotate in GENCODE, lncRNA fragments, pseudogenes, etc).

To examine whether tissue-elevated, small RNA sequences (comprised of miRNA, piRNA, snoRNA, snRNA, tRNA and YRNA fragments, etc) were found in specific biofluids and correlated with tissue abundances, we produced a high-quality tissue atlas of small RNAs across 30 human tissues. Tissue samples were received from the Brain and Body Donation Program (BBDP) at Banner Sun Health Research Institute (http://www.brainandbodydonationprogram.org); samples were rapidly collected with a median postmortem interval of 3 h and verified to be pathologically normal (see Methods; donor demographic information is available in Supplementary Table S2). Samples were sequenced deeply with at least 9.8 million reads mapping to miRNA. Supplementary Figure S1 displays rarefaction curves that have plateaued for new miRNA detection in each tissue. The three biological replicates were averaged together for each tissue, the human genome mapping rate was high for all tissues (Figure 2A). The RNA biotypes that make up the transcriptome alignments for each tissue are shown in Figure 2B. Gallbladder has high levels of tRFs and piRNA sequence alignments; whereas, monocytes and PBMCs display higher levels of yRFs, potentially contributing to the high abundance of yRFs in plasma samples.

### Detection and Classification of Tissue-Elevated Sequences

**FIGURE 3:**
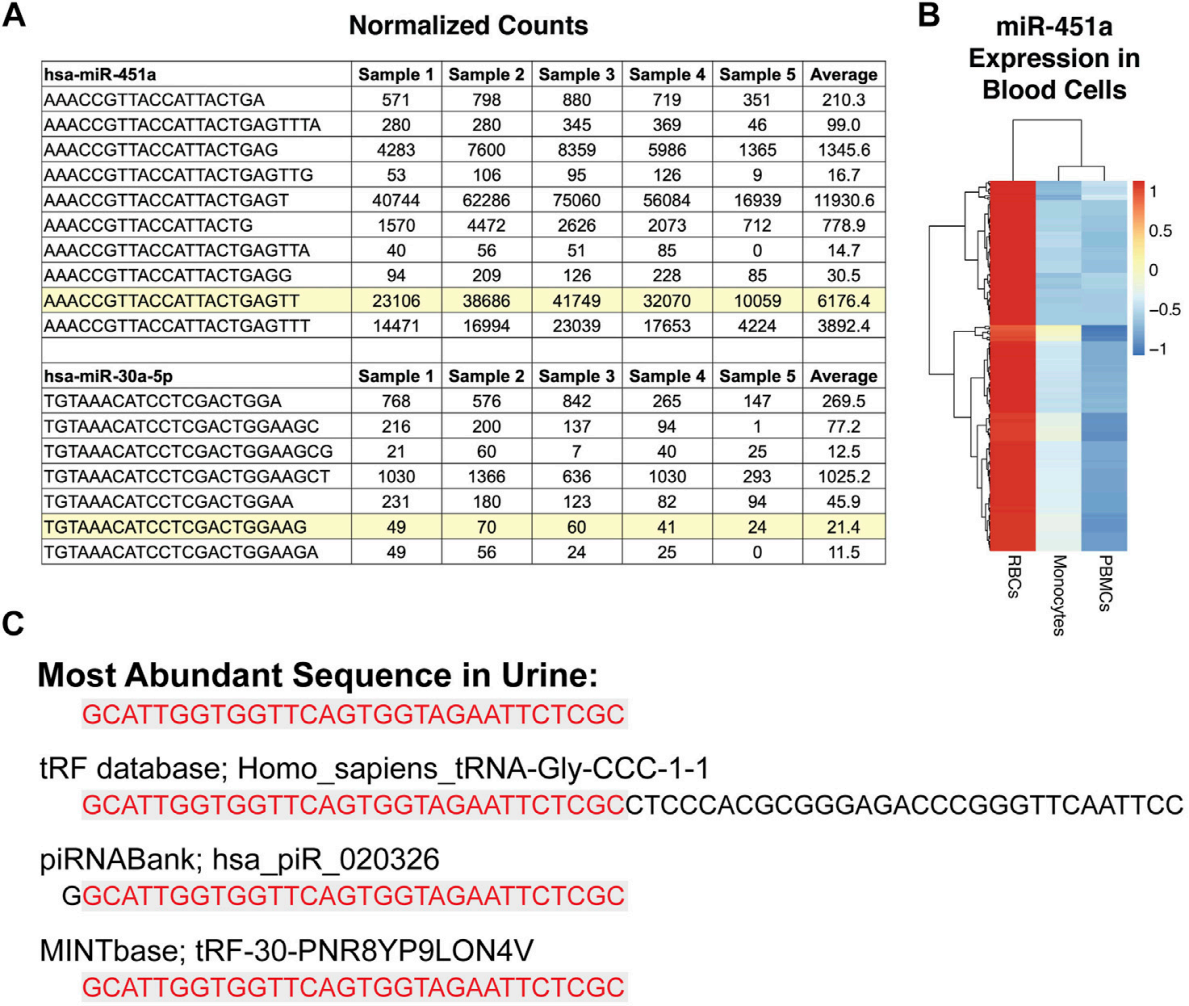
Sequence Variation and Categorization. Panel **(A)** highlights the canonical miRNA sequence that is entered in miRbase for two miRNAs, miR-451a and miR-30a-5p. The other sequences are isomiRs of the mature sequence (highlighted) listed in miRbase. The table shows the normalized expression for the sequences in plasma from five different subjects and the average across the 179 cell-free plasma samples. In some cases, an isomiR has higher expression in plasma than the highlighted canonical sequence. miR-451a is highly expressed in red blood cells, as can be seen in the heat map in Panel **(B)**. Panel **(B)** displays the canonical and isomiR sequences for miR-451a found in red blood cells, PBMCs, and monocytes. The most abundant sequence detected in urine samples is displayed in Figure 3c and is = similar to both a piRNA and a tRNA sequence.

There is overlap in the expression of canonical miRNAs between different tissues, as well as some unique tissue-elevated miRNAs. We sought to expand the number of tissue-elevated sequences by identifying abundant sequences that do not conform to canonical small RNA annotations. We performed a strict alignment of small RNA sequences from the biofluids and tissues using the exceRpt pipeline (see Materials and Methods). We used miR-451a and miR-30a-5p as examples to demonstrate the presence of the mature canonical sequences and the number of isomiRs present in plasma samples. There are 73 isomiRs of miR-451a with >1 read in >25% of plasma samples (Supplementary Table S3). The mature canonical sequence for miR-451a, highlighted in Figure 3A, is not the most abundant sequence (Guo and Chen, 2014; Liang et al., 2017). MiRNA miR-451a (Griffiths-Jones, 2004; Kozomara et al., 2019) is highly abundant in RBCs (Sun et al., 2020) and we used a heatmap to display expression (Figure 3B) of all miR-451a isomiRs in RBC, PBMCs and monocytes. miR-451a is a dicer-independent miRNA (Liang et al., 2017) while the second example in Figure 3A is from a dicer-dependent miRNA, miR-30a-5p. miR-30a-5p has 18 detectable isomiRs with >1 read in >25% of cell-free plasma samples with many of them more abundantly detected than the highlighted mature sequence found in miRBase.

The most abundant sequence in urine is GCA​TTG​GTG​GTT​CAG​TGG​TAG​AAT​TCT​CGC (Yeri et al., 2017), which matches a piRNA entry (hsa_piR_020326) with one less 5’ G, as well as being an exact match for a portion of a tRF (Figure 3C). Despite being an exact match, typical tRFs and those used by the tRF database in our alignment pipeline, exceRpt (Rozowsky et al., 2019), are longer than the sequence we detect (see red shading to indicate the detected fragment compared with the full-length fragment in the database). A recent paper introduced a tRF database called MINTbase (Wang et al., 2019), which describes and counts the sequence abundantly detected here in urine, as an exact match to a tRF (Figure 3C). It is important to keep these alignment and naming challenges in mind, as these sequences are short, and in many cases, cannot be classified with complete certainty back to the original parent RNA.

**FIGURE 4:**
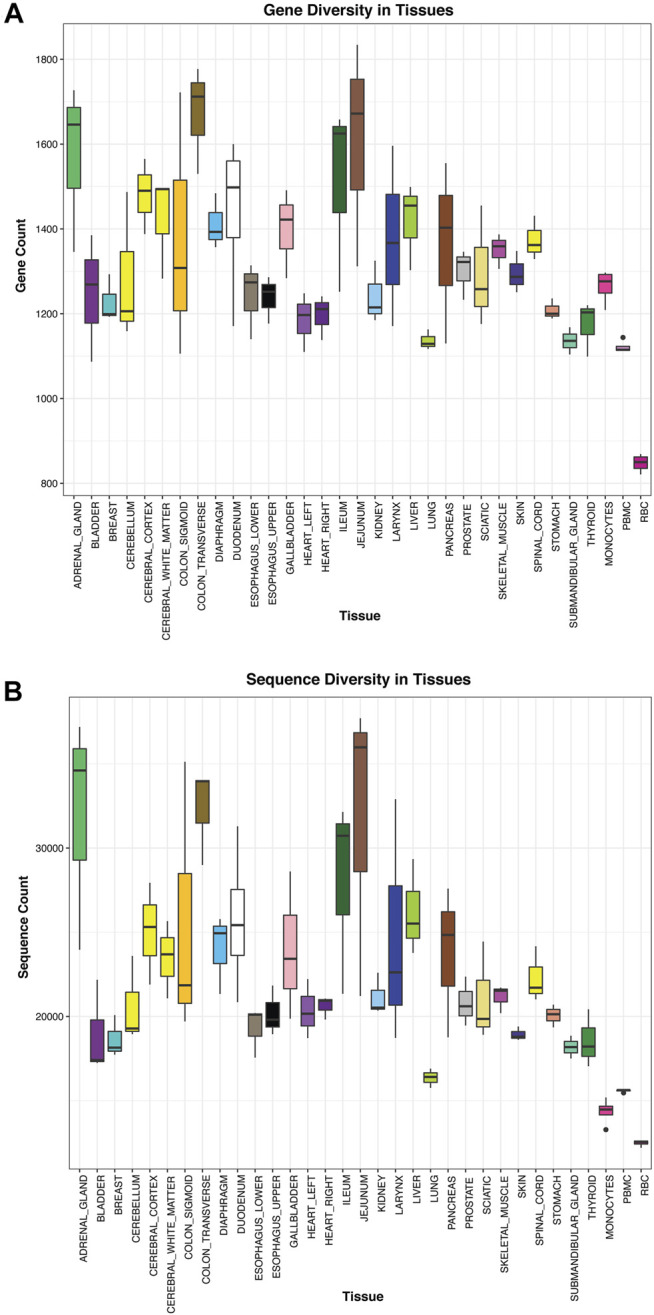
Tissue Diversity. Panel **(A)** displays the number of genes detected using small RNASeq in each tissue. In this case, all isomiRs, isoforms, and fragments are collapsed to the parent gene and counted once. In Panel **(B)**, all unique sequences, isomiRs, isoforms, and fragments are counted separately and summed up.

Using the tissue atlas of small RNAs that we generated, we next explored sequences for tissue-elevation. We generated a list of sequences at least seven-fold enriched in a maximum of four tissues using the R package, TissueEnrich (Jain and Tuteja, 2019). The TissueEnrich package contains three categories of “enrichment.” We employed tissue-elevated as the combination of the three categories (“tissue-enriched,” “group-enriched” and “tissue-enhanced”). The reason for four tissues was due in part to having multiple regions for a particular tissue (e.g., central nervous system - cerebellum, cerebral cortex, cerebral cortex-white matter, spinal cord). Tissue-elevated sequences and their fold-enrichments are available in Supplementary Table S4. Figure 4 describes the number of genes (4a) or individual sequences (4b) that were identified to be elevated in each tissue. Figure 4A represents the number of genes detected when all isomiRs and sequence fragments have been collapsed down to the parent/canonical gene. The sequences in Figure 4B represent individual isomiRs, and unique RNA fragments of genes that are expressed at a minimum of 25 counts in at least 2/3 biological replicate samples. The numbers of these tissue-elevated sequences that go to miRNA and piRNA for each tissue and biofluid are displayed in Table 1 and the full table with all RNA biotypes can be found in Supplementary Table S5. The graphs and tables can be used to determine that RBCs and monocytes, and tissues such as bladder and gall bladder, have fewer distinct tissue-defining sequences, while interestingly – jejunum – has many tissue-elevated sequences. Table 1 and Supplementary Table S5 describe the sequences detected in each tissue broken out by small RNA biotype. First column for each RNA biotype is the number of detected genes identified for each tissue (all isomiRs and similar fragments are collapsed and counted with the canonical sequence), followed by the full number of isomiRs or unique fragments (not collapsed; column two) for that RNA biotype, for each tissue. We also include the number of tissue-elevated genes (column three) and fragments/isoforms (column four) for each RNA biotype in each tissue. While the pattern of tissue-elevated sequences is similar in Figures 4A,B, the scales are very different. For example, adrenal gland has 1,572 tissue-elevated genes when collapsed (Figure 4A) and has 31,917 tissue-elevated unique sequences with >10 reads in at least 2 of the three replicate tissue samples (Figure 4B). We also observe low small RNA diversity in monocytes and RBCs.

**TABLE 1:** Tissue-Enriched Sequences. Displays the # of select RNA biotypes detected, the number of isoforms detected, and the number of tissue-enriched sequences observed for each tissue.

Sample	miRNA	isomiRs	Tissue enriched miRNA	Tissue Enriched isomiRs	piRNA	piRNA Isoforms	Tissue Enriched piRNAs	Tissue Enriched piRNA isoforms
Adrenal gland	474	11737	71	554	149	3644	85	1086
Bladder	485	12171	36	65	131	1550	1	1
Breast	465	11676	9	88	132	1234	5	16
Cerebellum	523	14077	137	2141	131	1087	4	4
Cerebral cortex	551	14224	183	2926	142	1571	19	38
Cerebral - white matter	543	13073	111	1698	142	1734	9	13
Colon – sigmoid	464	12638	28	85	142	2998	26	81
Colon - transverse	474	12239	32	419	142	3344	74	1183
Diaphragm	542	13319	57	532	140	1997	6	11
Duodenum	475	12578	19	112	141	2596	3	4
Esophagus - lower	467	11962	6	306	134	1261	3	4
Esophagus - upper	465	11724	11	274	135	1651	1	1
Gallbladder	481	12170	67	173	138	2724	58	819
Heart – left	486	12817	34	320	131	1117	2	6
Heart – right	496	12965	44	390	130	1080	5	17
Ileum	457	11570	10	128	145	3415	41	168
Jejunum	475	11237	13	413	142	3269	44	122
Kidney	455	11255	34	244	135	1371	1	2
Larynx	496	13973	39	446	142	2639	31	172
Liver	436	10343	21	575	142	2585	21	63
Lung	461	11292	12	15	126	893	6	8
Pancreas	470	12084	29	404	140	2543	18	38
Prostate	473	13103	85	493	136	1544	12	47
Sciatic	487	11773	10	22	136	1923	2	3
Skeletal muscle	524	14110	97	1940	134	1107	6	10
Skin	466	10860	16	95	135	1270	4	6
Spinal cord	531	12213	59	928	137	1443	9	18
Stomach	448	12597	12	90	132	1596	2	4
Submandibular gland	451	11663	23	503	130	981	0	0
Thyroid	448	11235	12	114	133	1415	0	0
Monoctyes	455	9496	188	1519	119	984	35	79
PBMC	443	9867	79	1323	112	659	14	24
RBC	403	9967	225	3656	73	274	8	11
CSF	122	610	49	136	24	259	14	116
PLASMA	357	5863	152	913	52	281	13	38
SERUM	180	1714	40	177	71	1030	33	129
SALIVA	240	3200	80	503	46	729	26	222
URINE	155	1684	46	131	53	2594	31	377
PLASMA_EVs	381	8069	202	1374	43	297	15	40

**FIGURE 5:**
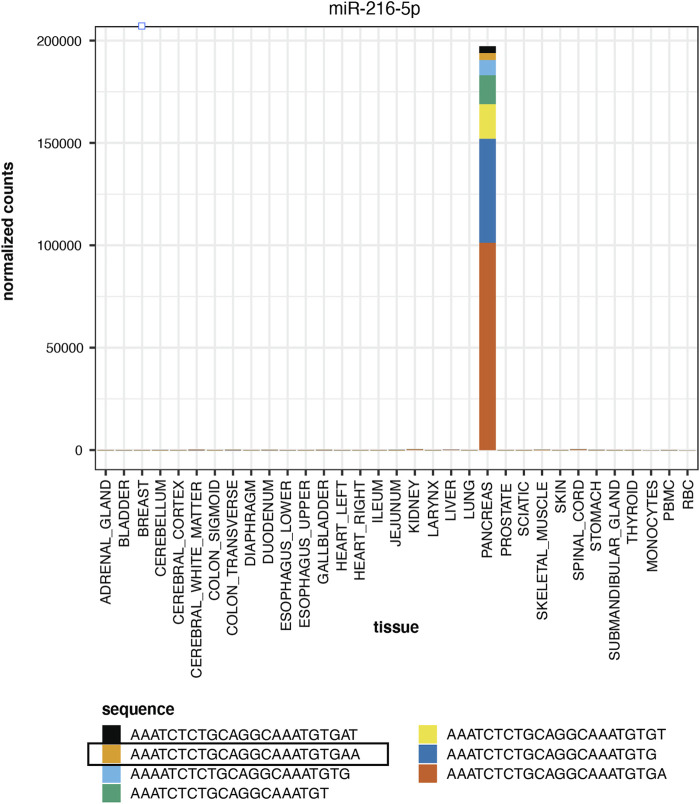
Tissue Elevation. miR-216-5p is elevated in pancreas. The expression of the mature sequence is highlighted in Panel. The stacked bar plot displays the relative proportion of other miR-216-5p isomiRs, all of which are elevated in pancreas.

Figure 5 displays the normalized counts for a pancreas-elevated miRNA, miR-216-5p, as well as the normalized counts for miR-216-5p and its isomiRs in all tissues. The canonical sequence has 50% of the reads and the other 50% of miR-216-5p reads are distributed to its 6 isomiRs. All of these sequences are elevated in pancreas, compared to other tissues.

### Tissue-Enriched miRNAs Cluster in Tissues and Blood Cell Types

**FIGURE 6:**
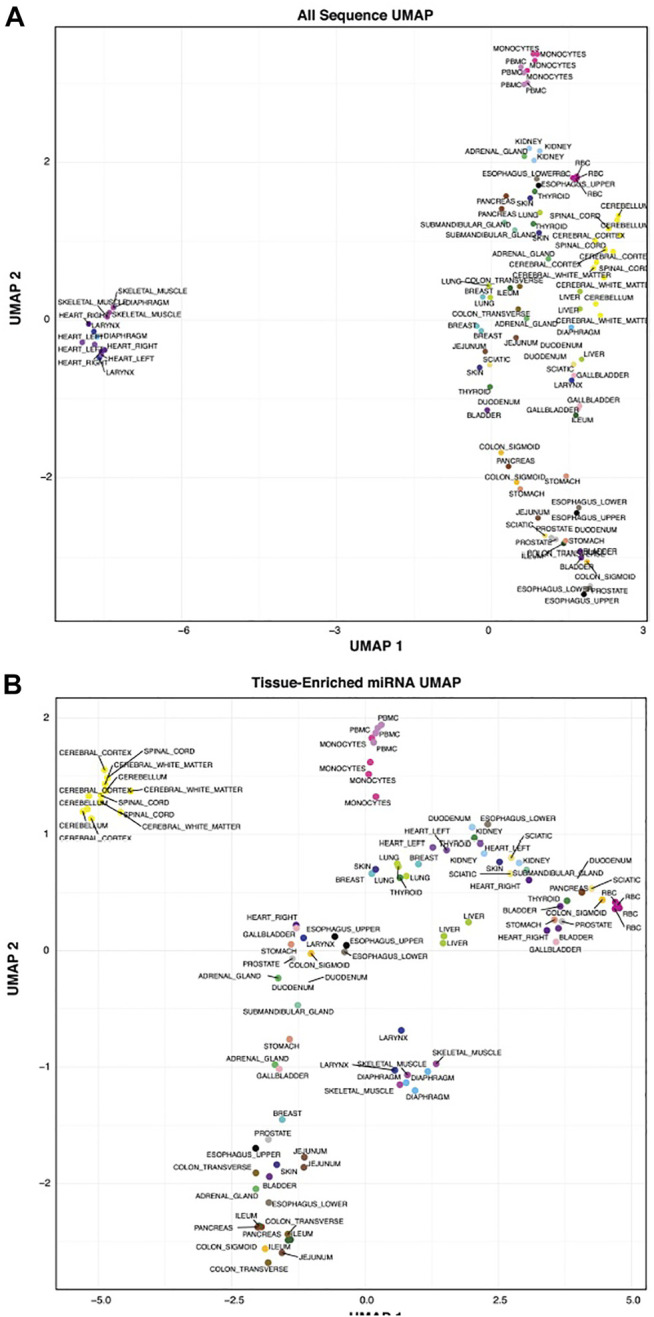
Tissue Clustering. We clustered the tissues using all sequences in Panel **(A)**. Using tissue-elevated miRNA sequences in Panel **(B)**, the tissue samples clustered into tighter groups. In both figures, the tissue replicates show low variability.

We wanted to determine how well tissues clustered based on their related sequences. Figure 6A is a UMAP using all sequences to cluster the tissues and blood cells and Figure 6B UMAP is subset to using tissue-elevated miRNA and isomiR sequences from each tissue for clustering. Most tissue replicates show consistency and cluster closely, regardless of the sequences used to generate them. Using only the tissue-elevated sequences improved clustering in the CNS tissues. Supplementary Figure S2 displays tissue clustering using the tissue-enriched tRFs, yRFs and piRNAs, each of which display less tissue specificity than tissue-elevated miRNAs/isomiRs and greater variability among tissue replicates.

### Tissue-Elevated Sequences are Detected in Biofluids

**FIGURE 7:**
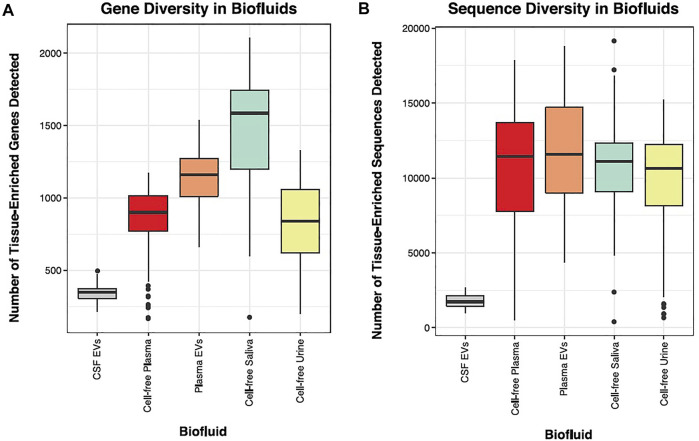
Biofluid Diversity. Panels **(A, B)** display the number of tissue-enriched genes identified in the small RNA sequenced data. The parent tissue-elevated genes that were detected in each biofluid are displayed in Panel **(A)**. CSF had the lowest number of tissue-elevated genes and saliva had the highest. Panel **(B)** displays the number of tissue-elevated sequences that were detected in each biofluid.

We sequenced 30 tissues, representing most major organ systems in the body and classified tissue-elevated small RNA sequences. We next wanted to measure the number of tissue-elevated genes and sequences that were detectable in biofluids: CSF-EVs, plasma-EVs, cell-free plasma, cell-free saliva, and cell-free urine. Figure 7 displays the diversity of tissue-elevated genes (Figure 7A) and sequences (Figure 7B, including isomiRs and RNA fragments) detected in each biofluid. Table 1 provides the number of tissue-enriched sequences, taken from the tissue atlas portion of the paper, and present in each biofluid (Supplementary Table S5 provides the data for all RNA biotypes). Plasma samples contain the largest number of tissue-elevated sequences compared to the other biofluids.

**FIGURE 8:**
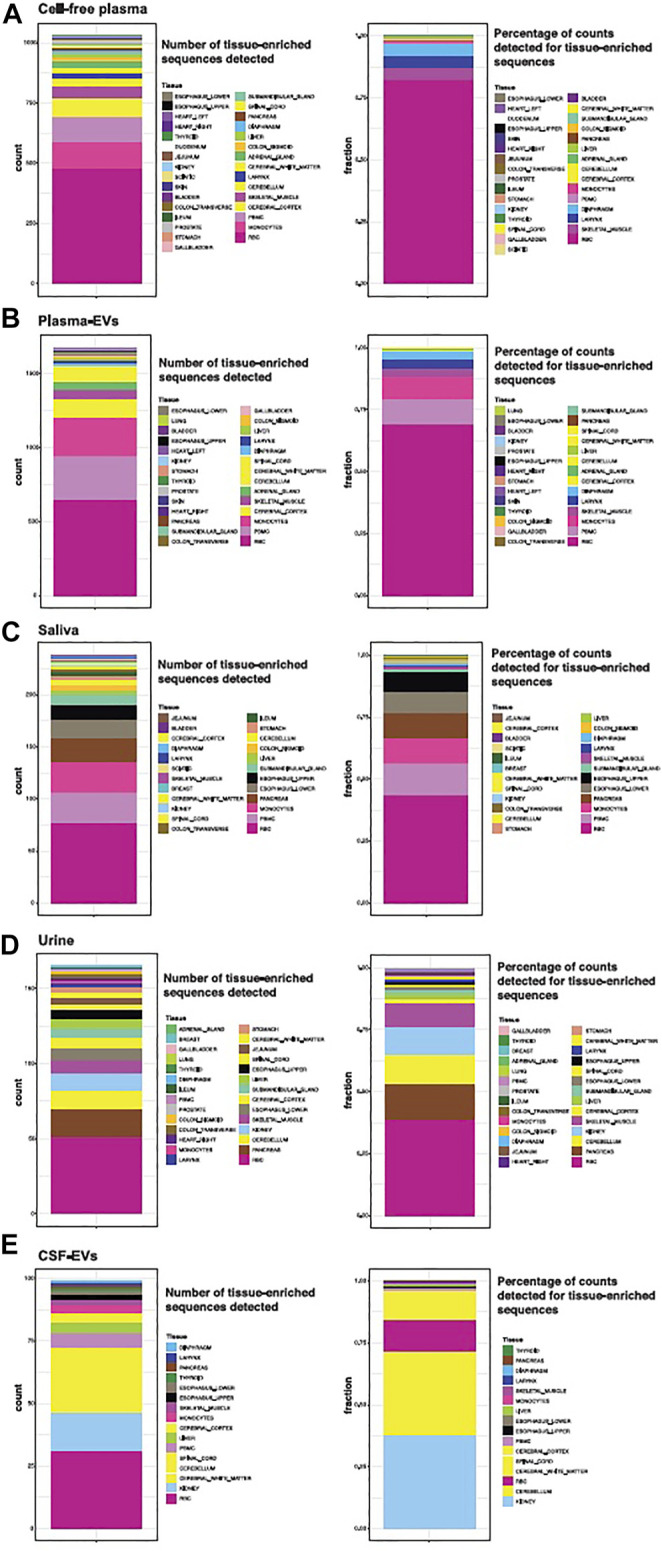
Tissue-elevated miRNA/isomiR Sequence Detection in Biofluids. The number of tissue-elevated sequences that were detected in each biofluid and their abundance are displayed in Panel. The number of tissue-elevated sequences detected for each tissue in cell-free plasma are shown in the first stacked bar plot Panel **(A)**. Tissue-elevated sequences from 31 different tissue and cell types were detected (the numbers are found in Supplementary Table S5). The second stacked bar plot in Panel **(A)** displays the fraction of tissue-elevated reads going to each sample type. 69% of the tissue-elevated reads in cell-free plasma go to red blood cells. The number of tissue-elevated sequences detected and the fraction of tissue-elevated reads going to each tissue are displayed for plasma EVs [Panel **(B)**], saliva [Panel **(C)**], Urine [Panel **(D)**], and CSF [Panel **(E)**].

We looked more closely at the number of detectable tissue-elevated sequences in cell-free plasma, plasma-EVs, cell-free saliva, cell-free urine, and CSF-EVs (Figure 8). We examined tissue-elevated miRNAs and isomiRs, since they are the most diverse sequences and clustered tissues well in Figure 6B. In each panel, Figures 8A–E, the first stacked bar plot represents the number of tissue-enriched miRNA and isomiR sequences detected in that biofluid with >10 counts in >50% of samples, and the second stacked bar plot is the sum of all tissue-elevated counts for each tissue. If two tissues share a tissue-elevated sequence, the sequences are counted for both tissues. For Figure 8, the number of tissue-elevated sequences detected in each biofluid are provided in Supplementary Table S6, the number of tissue-elevated sequences and the fraction of the reads they account for in each biofluid are provided. Cell-free plasma contained tissue-elevated sequences from the largest number of tissues and CSF from the least number of tissues. There are 474 tissue-enriched RBC miRNA/isomiR sequences found in cell-free plasma, accounting for an average of 82% (874,975 counts) of the tissue-enriched sequences detected in cell-free plasma. Saliva samples display high levels of RBC sequences as well as sequences from esophagus and submandibular gland (Figure 8C). Interestingly, pancreas-enriched sequences are also found in high numbers. Kidney and brain tissue-enriched sequences tended to overlap, and both urine and CSF display high levels of kidney and CNS tissue-elevated sequences. The two sequences most abundantly detected in CSF are tissue-elevated for both cerebellum and kidney, the canonical sequence for miR-204 and an isomiR-204, and are counted for both tissues. Tissue-elevated sequence abundance from RBCs make up the majority of the overall number of counts in plasma-EVs. 3% of the tissue-enriched sequences in plasma-EVs were from skeletal muscle and other tissues that cluster with skeletal muscle in the UMAP in Figure 6B. We would like to point out caveats to our approach: 1) tissue-elevation does not equal tissue-specificity, sequences can be associated with and expressed in more than one tissue, 2) we did not sequence every tissue or cell type in the body, 3) each tissue has a different number of elevated sequences, tissues with fewer sequences were not weighted differently from tissues with a large number of sequences, and 4) tissue vascularity and size were not accounted for.

tRFs illustrate one area of caution when examining these data. Forty-six tissue-elevated tRF sequences were detected in cell-free plasma, 14 of which were tissue enriched in gallbladder. Gallbladder had a high level of tRFs compared to other tissues (Figure 2). The tRF with the sequence “TCC​CTG​GTG​GTC​TAG​TGG​TTA​GGA​TTC​GGC​GC” comprises 10.2% of gallbladder tissue small RNASeq reads. Because it is so highly expressed in the gallbladder, it is counted as ‘elevated’ in gallbladder by the criteria we used in this paper. It is nearly 10-fold more highly expressed in gallbladder than in the next most abundant tissue, Ileum. However, as can be seen in Table 2, this tRF is highly expressed in most tissues, but low in blood cell types. This illustrates that while this gallbladder-elevated tRF is detectable in every biofluid we examined, it is high expression in all tissues makes it a poor marker of tissue specificity.

**TABLE 2:** Tissue-enriched gallbladder tRF. A tRF sequence that is enriched in gallbladder is also highly expressed in many tissues.

Tissue	Normalized counts
ADRENAL_GLAND	120,004
BLADDER	127,655
BREAST	44,534
CEREBELLUM	124,302
CEREBRAL_CORTEX	137,064
CEREBRAL_WHITE_MATTER	424,818
COLON_TRANSVERSE	75,749
DIAPHRAGM	381,206
DUODENUM	619,992
GALLBLADDER	15,953,357
ILEUM	1,615,212
JEJUNUM	284,800
KIDNEY	139,955
LARYNX	958,023
LEFT_HEART	22,264
LIVER	530,800
LOWER_ESOPHAGUS	58,251
LUNG	66,785
MONO	11,612
PANCREAS	187,907
PBMC	6,166
PROSTATE	64,484
RBC	490
RIGHT_HEART	22,535
SCIATIC	393,225
SIGMOID_COLON	114,936
SKELETAL_MUSCLE	34,388
SKIN	136,642
SPINAL_CORD	164,628
STOMACH	164,674
SUBMANDIBULAR_GLAND	37,790
THYROID	146,499
UPPER_ESOPHAGUS	121,985

### Circulating, Tissue-Elevated Small RNAs are Differentially Expressed in Physiological (Exercise) and Pathologic States (Liver Fibrosis or Ischemic Stroke)

**FIGURE 9:**
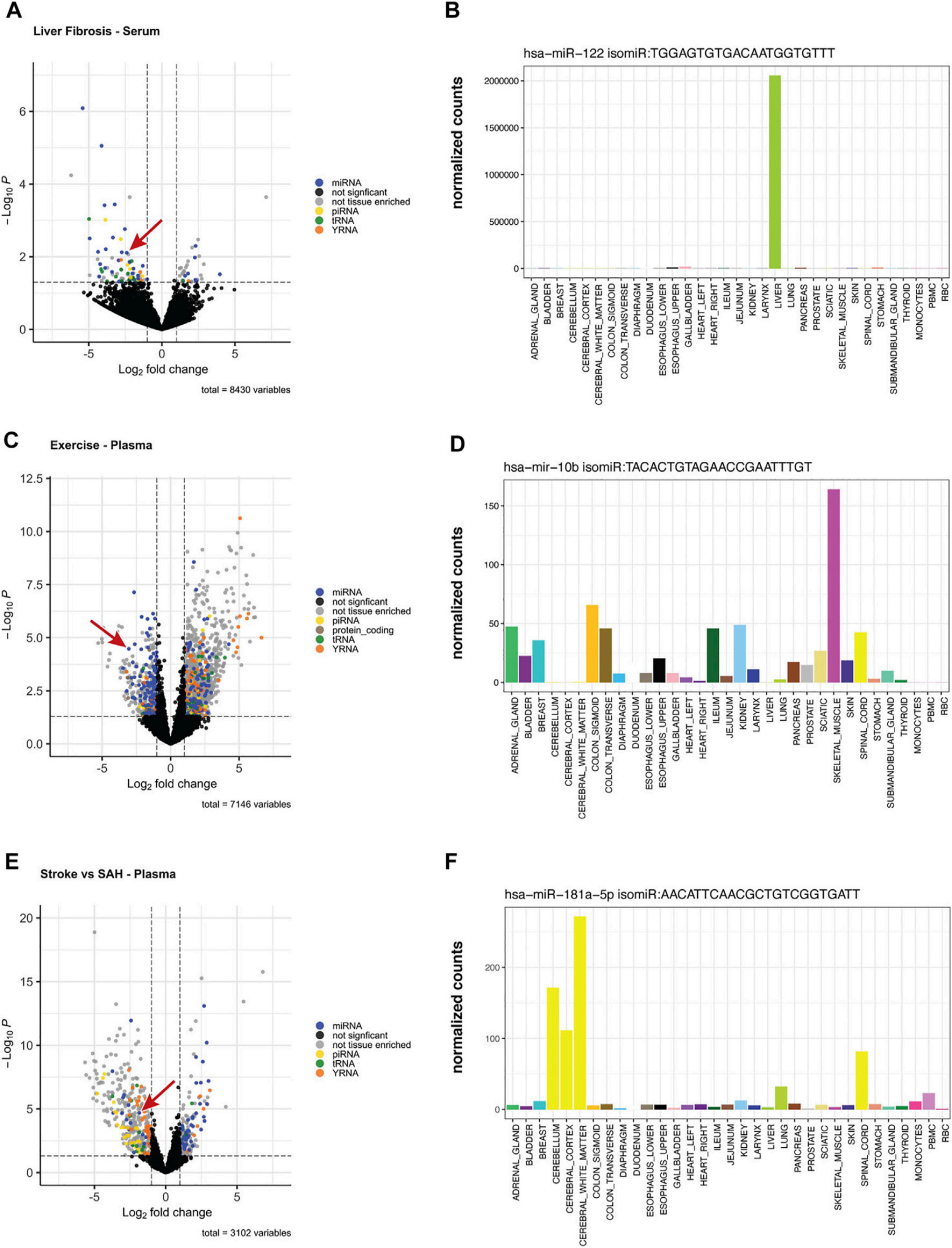
Differentially Expressed Tissue-Elevated Sequences. We examined three different conditions for differentially expressed sequences: **(A)** displays the differentially expressed tissue-elevated sequences found in blood samples from Hepatitis C patients with liver fibrosis stage 1 or stage 4. Panel **(B)** is a tissue-elevated isomiR that is highly expressed in liver. **(C)** are samples taken from participants before and after exercise, and the expression level of a muscle-elevated isomiR across tissues **(D)**. The final example is from a comparison of plasma samples taken from individuals that had either an ischaemic stroke or a subarachnoid hemorrhage, Panel **(E)**. In Panel **(F)**, we selected a CNS-elevated isomiR of miR-181-5p to illustrate tissue-elevation.

To assess the potential for tissue-elevated isomiRs to be differentially expressed in physiological or pathological states, we re-examined previously published data from three different studies. The first study compared blood samples from subjects with Hepatitis C and liver fibrosis stage 1 (early) and liver fibrosis stage 4 (advanced) (Van Keuren‐Jensen et al., 2016). Figure 9A shows the number of differentially expressed and tissue-elevated sequences and their RNA biotypes identified in blood samples. miRNAs or isomiRs that were tissue-elevated, in any tissue, are blue dots in the volcano plot, tissue-elevated piRNAs or sequence isoforms are yellow dots, tissue-elevated tRNA sequences are green, tissue-elevated YRNA sequences are orange, and sequences that were differentially expressed but not tissue-elevated are gray. Sequences that do not make the cutoff for *p*-value or fold-change are black. These RNA biotypes represented the largest number of differentially expressed sequences. We focused on a tissue-elevated isomiR in Figure 9A (miR-122 isomiR, TGG​AGT​GTG​ACA​ATG​GTG​TTT), indicated by the red arrow. The expression level of this isomiR in tissues is displayed in Figure 9B. This miR-122 isomiR, like the canonical miRNA, is liver elevated and expressed very highly.

Towards employing the tool to distinguish sequences differences in a physiological example, we reanalyzed changes in miRNA detection in plasma samples pre- and post-exercise. Samples were taken from 26 participants at baseline and immediately after peak exercise using the Bruce Treadmill test. Using all unique sequences and isomiRs, we compared baseline expression to expression immediately after exercise (Shah et al., 2017). Figure 9C displays the number of tissue-elevated sequences following exercise. An isomiR of miR-10b, which is tissue-elevated in skeletal muscle was significantly down-regulated following exercise, Figure 9D.

In the final dataset, we examined plasma samples from patients with two types of stroke, either ischaemic stroke (cerebral vessel occlusion) or a subarachnoid hemorrhage. We examined the differentially expressed sequences that may be able to differentiate these two types of stroke and did differential expression analysis of plasma samples. From our differentially expressed sequences, we focused on a CNS tissue-elevated sequence, an isomiR of miR-181-5p, that was downregulated in stroke and upregulated in subarachnoid hemorrhage, Figure 9E. In Figure 9F, you can see the elevation of this sequence across the CNS tissues.

### The exRNA Expression Atlas—an Online Tool to Display Tissue-Elevated Sequences in Biofluids

**FIGURE 10:**
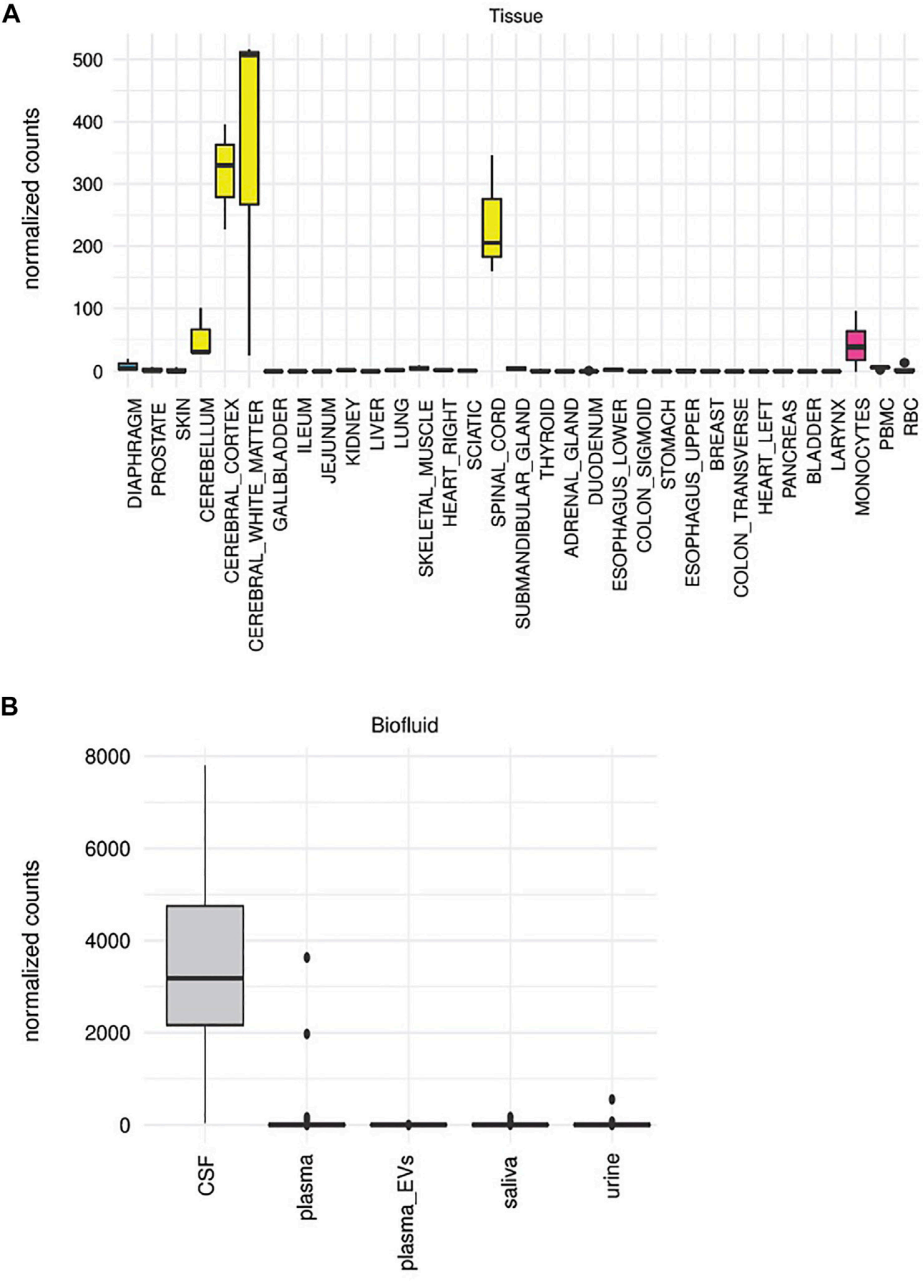
exRNA Expression Atlas. In **(A)** we display the expression of a tissue-elevated miRNA for the CNS (miR-1298) across all tissues. Panel **(B)** displays the detection of tissue-elevated miR-1298 in different biofluids. As can be seen in this figure, this enriched CNS miRNA is detectable in CSF, but poorly detected in the other biofluids. These data will provide insight regarding which biofluids hold which tissue-elevated sequences.

To facilitate the widest utility of this data, we created a searchable, online tool with tissue-elevated sequences, and the biofluids in which they are present. Figure 10 displays output capabilities of the tool, the exRNA Expression Atlas (https://data.omics.kitchen/miRNAatlas/). To illustrate how this tool works, we chose miR-1298 as an example of a miRNA that is highly enriched for tissues of the central nervous system [Figure 10A; (Godoy et al., 2018)]. Figure 10B displays the biofluids in which this miRNA can be detected. miR-1298 is most highly detected in CSF with minimal counts in other biofluids for most subjects. Knowing the expression of a miRNA such as this one in tissues, and its detection in CSF, may help prioritize biomarker candidates. As such, this searchable, online tool may assist in the validation of studies aiming to demonstrate tissue-specificity for RNA species.

## Discussion

Small RNA databases and resources currently exist, but are subject to confounders, such as a lack of overlapping tissues in each dataset, differential RNA isolations that can lead to potential batch effects, and inconsistent sequencing/alignment of the reads across studies. To address these unmet needs, we created a tissue atlas of small RNAs in 30 human tissues with three replicates and multiple blood cell types. Strengths of our study are the inclusion of pathologically normal tissue with low postmortem interval and uniformity in RNA isolation and sequencing and bioinformatic handling. We found that tissues had varying percentages of RNA biotypes and diversity of tissue-elevated sequences. One recent paper that has similarly brought together these disparate resources is by Rahman *et al.* (Rahman et al., 2020), where the investigators examined small RNA biotypes from different tissues to come up with a set that they found to be tissue-enriched.

Using tissue-elevated sequences, especially miRNAs, we clustered the tissue samples with low variability among biological tissue replicates. Tissue-elevated tRFs and yRFs successfully clustered the samples, despite greater variability among replicates. Surprisingly, as a category, the number of tissue-elevated tRF sequences were high for several tissues: gallbladder has 2283 tissue-enriched tRFs, adrenal gland has 3805 tissue-enriched tRFs, and transverse colon has 3782 tissue-enriched tRFs. The use of all unique sequences provided us with interesting observations about some of the tissue-elevated sequences. For example, by expanding the miRNA biotype to include all isomiRs, we found a heart enriched miRNA that was more highly expressed in right heart than the left heart. These data provided us with an opportunity to use these tissue-elevated sequences to explore their detection in biofluids. One qualification for this dataset is that the RNA from the tissue samples was isolated and prepped uniformly, however different RNA isolation and sample preparation protocols, optimized for low input RNA, were used for the cell-free biofluids and EVs. A second qualification for this dataset is the use of postmortem tissues from older individuals, while most of the biofluid samples are from much younger subjects. It is known that small RNA profiles can change with age (Fehlmann et al., 2020).

There are several previously published approaches to deconvolute the origin of specific RNAs from a varied set of tissue types, but this has not been widely extended to biofluids or EVs. In this case, we decided not to do deconvolution for examining the tissue contributions in biofluids, given the lack of known correspondence between enrichment of sequences in a given tissue with the release of EVs bearing that RNA cargo from that tissue into biofluids. Instead, we relied on a more “direct” approach to measure the diversity and abundance of tissue-elevated sequences detected in each biofluid based on the total frequency of tissue-elevated sequences detected in each biofluid. A unique finding of this approach is the presence of similar transcripts across biologically divergent tissue types. For example, renal and brain RNAs were abundant in samples from the CSF. The biological etiology of this is elusive, but is certainly plausible given the similar physiologic function of filtration and electrolyte regulation in both urine and CSF. The two sequences with the highest detection in CSF are tissue-enriched for both kidney and cerebellum and are detected with >1000 counts each in CSF, miR-204-5p and an isomiR of that miRNA.

In the examination of differentially expressed tissue-elevated sequences found in blood, it was interesting to note that we were able to identify tissue sequences related to the disease or event. Because we do not have corresponding tissue samples from these individuals, it is hard to know if these changes directly correlate with the tissue, or if there is different usage or packaging of these sequences into the extracellular biofluid.

Clinically, this simplistic “deconvolution” within biofluids may facilitate targeting those RNAs that may be more specific for disease monitoring. Certainly, this tissue-enrichment estimation is imperfect. First, we do not have many replicate tissues and samples from every tissue or cell type within that tissue. Nevertheless, we believe that the current study provides a framework to build shared resources to assist in studies of tissue- and cell-specific RNAs and their presence in biofluids (exRNAs). Some obvious confounding factors to target in future experimental designs include: 1) some tissues may actively release more EVs or EVs with more/specific RNA cargo, 2) vascularization of the tissue may lead to greater abundance in plasma, and 3) selective RNA loading that does not contain tissue enriched sequences for some of the tissues will lead to the appearance of low abundance in plasma, and 4) understanding expression level changes with disease, injury, stress, development or aging. In addition, care to avoid overinterpretation of “concentration” of a given exRNA species is needed: for example, the abundant gallbladder-enriched tRF is also abundant across many other tissues. Ultimately, with the incorporation of larger numbers of matched tissue and biofluid samples, these data may expand the repertoire of disease-relevant exRNA biomarkers. We have created a tool (the exRNA Expression Atlas) to display tissue-elevated sequences and their abundance in biofluids that is freely accessible to the scientific community.

## Data Availability

All sequencing data is available through the ERCC exRNA Atlas: https://exrna-atlas.org/exat/datasets (EXR-KJENS1WBaSro-AN), https://exrna-atlas.org/exat/datasets (EXRKJENS1sPlvS2AN), https://exrna-atlas.org/exat/datasets (EXR-KJENS1sPlvS2-AN), https://exrna-atlas.org/exat/datasets (EXR-KJENS1TISSUEDATA-AN); and through accession number phs001258.v1.p1 in dbGaP: https://www.ncbi.nlm.nih.gov/gap/.

## Supplementary Material

Supplementary Figure S1 This graph shows the rarefaction curves of each tissue. As can be seen here, the detection rate of new miRNAs rises steeply and then begins to plateau at 1 million reads and 3 million reads. These data indicate that using the most diverse class of small RNA, we are capturing most of the diversity for each tissue with our read depth.

Supplementary Figure S2 Supplemental figure 1 displays the UMAP clusters when using either tissue-elevated tRFs, piRNAs, or yRFs. tRFs do a better job of clustering tissues than do the other RNA biotypes displayed here. These sequences are not as tissue-elevated and the replicates are not as close as they are with tissue-elevated miRNA and isomiRs.

Supplementary Table S1 RIN values for tissue samples.

Supplementary Table S2 Demographic information for tissue samples.

Supplementary Table S3 IsomiR sequences for miR-451a in monocytes, PBMCs and RBCs.

Supplementary Table S4 Tissue-elevated sequences and their fold-enrichments.

Supplementary Table S5 The number of tissue-elevated sequences that go to each RNA biotype for each tissue and biofluid.

Supplementary Table S6 The number of tissue-elevated sequences detected in each biofluid.
