# Supplementary material for: A Novel Tissue Atlas and Online Tool for the Interrogation of Small RNA Expression in Human Tissues and Biofluids
Source: Front Cell Dev Biol. 2022 Mar 4;10:804164. doi: 10.3389/fcell.2022.804164 (PMC8934391; doi:10.3389/fcell.2022.804164)

Supplemental Figure 1

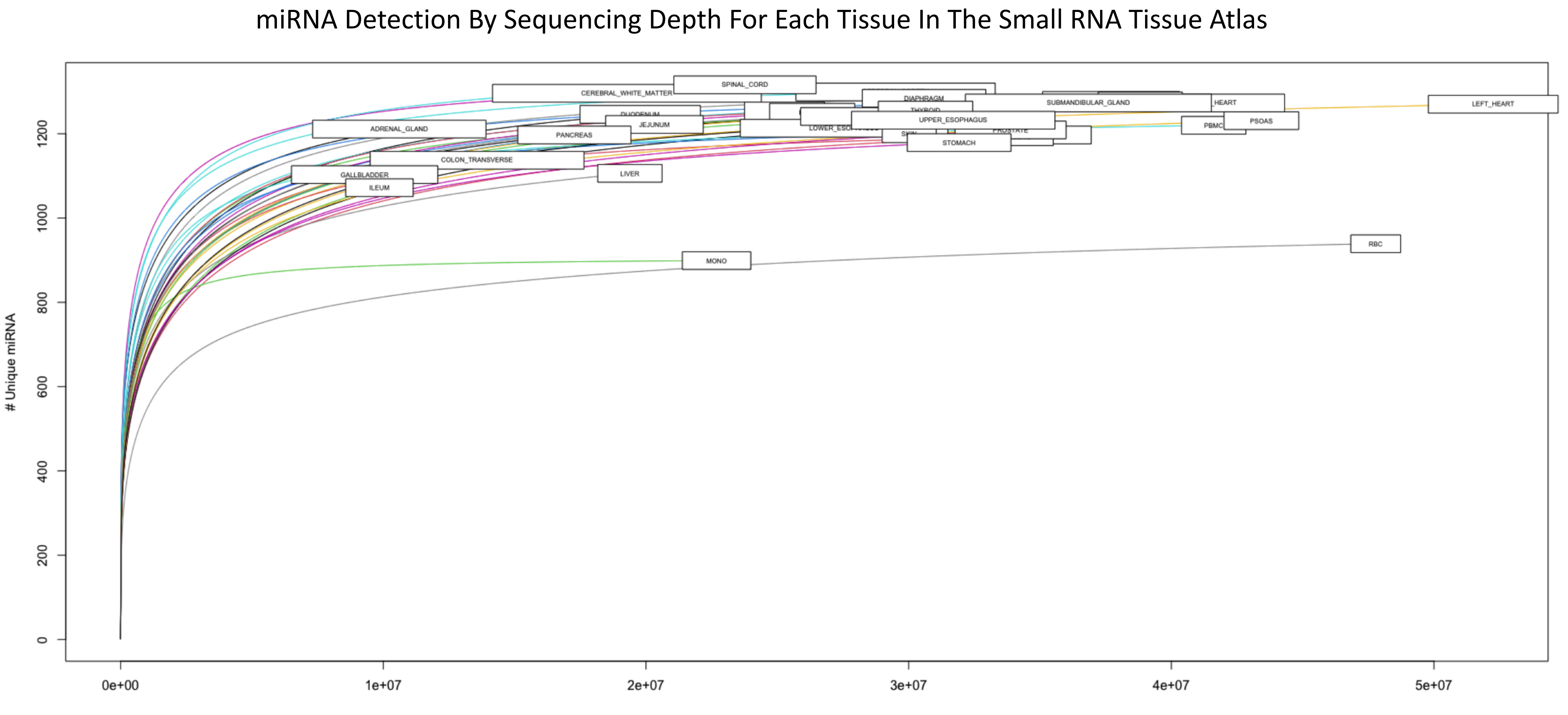

Supplement: Supplementary file 3 [file Image1.pdf]
